# Neurovascular dysfunction, ApoE and innate immunity: co‐conspirators in ARIA

**DOI:** 10.1002/alz.085572

**Published:** 2025-01-03

**Authors:** Costantino Iadecola

**Affiliations:** ^1^ Weill Cornell Medicine, New York, NY USA

## Abstract

Amyloid related imaging abnormalities (ARIA) are side effects of anti‐Abeta immunotherapy, which are most frequent and associated with greater morbidity in ApoE4 individuals. ARIA are characterized by neurovascular inflammation, leading either to increased vascular permeability and edema (ARIA‐E), or to more severe vascular damage and microhemorrhages (ARIA‐H). The mechanisms by which Abeta immunotherapy leads to ARIA remain to be established but may involve overload of the cerebral microvasculature by Abeta released from amyloid plaques. Abeta has profound proinflammatory and vasotoxic effects that alter the ability of cerebral blood vessels to regulate blood flow and damage the cellular constituents of the vessel wall. Using animal models of amyloid accumulation (tg2576 mice), we found that the deleterious neurovascular effects of Abeta are mediated by reactive oxygen species generated by perivascular and leptomeningeal macrophages, brain‐resident innate immune cells distinct from microglia and closely associated with cerebral arterioles and veins. The molecular mechanisms of the effect involve Abeta engaging the innate immunity receptor CD36 on these macrophages leading to free radical production by the enzyme NOX2. Accordingly, depletion of perivascular and leptomeningeal macrophages or deletion of CD36 or NOX in these cells prevents in full the damaging neurovascular effects of Abeta. Importantly, lack of CD36 also reduces Abeta accumulation in cerebral arterioles (cerebral amyloid angiopathy), a beneficial effect mediated by the ability of CD36‐deficient cerebral blood vessels to clear Abeta more effectively. Interestingly, brain macrophages also produce large amounts of ApoE, and in mice expressing human ApoE4 (ApoE4‐TR) ApoE4 in these macrophages triggers vascular oxidative stress and neurovascular dysfunction. Importantly, switching the genotype of the macrophages from ApoE4 to ApoE3, completely reverses the neurovascular dysfunction. These observations, collectively, point to brain resident macrophages associated with the cerebral microvasculature as key effectors of the neurovascular inflammation, oxidative stress, dysfunction and damage produced by Abeta. Inasmuch as ARIA are related to Abeta overload of the neurovasculature, targeting ApoE4, CD36 or NOX2 in these cells may be a putative therapeutic approach to prevent this deleterious vascular complication of Abeta immunotherapy.

